# Curriculum Innovations: High-Fidelity Simulation of Acute Neurology Enhances Rising Resident Confidence: Results From a Multicohort Study

**DOI:** 10.1212/NE9.0000000000200022

**Published:** 2022-11-15

**Authors:** Zahari N. Tchopev, Alexis E. Nelson, John C. Hunninghake, Kelsey Cacic, Melissa K. Cook, Morgan C. Jordan

**Affiliations:** From the Department of Neurology (Z.N.T., A.E.N., K.C., M.K.C., M.C.J.) and Department of Critical Care Medicine (J.C.H.), Brooke Army Medical Center, Ft. Sam-Houston, TX.

## Abstract

**Introduction & Problem Statement:**

The matriculation from internal medicine to neurology residency can be challenging. The most cogent approach to address this transition has yet to be identified. Studies show that simulation is highly effective at reinforcing knowledge and skills while improving learner confidence. We present the design and outcomes of an annual acute neurology simulation program.

**Objectives:**

We hypothesized that incoming neurology residents would (1) report improved confidence with managing acute neurologic emergencies, (2) cite a high degree of educational value with the program, and (3) demonstrate improvement in their technical knowledge.

**Methods & Curriculum Description:**

Our military, level 1 trauma institution's simulation laboratory and staff were used to develop and execute simulations for rising neurology residents based on the Accreditation Council for Graduate Medical Education neurology milestones. Three simulations were designed including a case of acute ischemic stroke (AIS), status epilepticus (SE) in an austere environment, and brain death evaluation with family counseling. Residents completed matched pre‐ and post assessments to gauge confidence, technical knowledge, and perceived educational value.

**Results & Assessment Data:**

Over 3 years, 15 rising neurology residents from 2 training programs completed 3 high-fidelity acute neurology cases. Self-reported confidence with acute neurology skills improved after each simulation. Confidence ratings included assessing for and identifying contraindications to tissue plasminogen activator, identifying AIS, identifying clot retrieval candidates, identifying clinical and electrographic SE, diagnosing and treating SE, identifying contraindications to and confounders of brain death diagnosis, performing the examination, and delivering bad news to families (all *p* < 0.05). Technical knowledge also statistically improved in the stroke (*p* = 0.046) and brain death simulation (*p* = 0.039), but not the SE simulation (*p* = 0.296). Participants reported an average perceived personal value of 4.8, 4.3, and 4.7 (out of a maximum of 5) for AIS, SE, and brain death simulations, respectively.

**Discussion & Lessons Learned:**

High-fidelity simulation of neurologic emergencies enhances confidence and knowledge of rising neurology residents. Satisfaction with the simulation cases was high. Academic hospitals can consider incorporating acute neurology simulations into their residency training.

Matriculating from internal medicine internship to neurology residency presents several challenges for the residents and their faculty. Residents need to be ready to rapidly and accurately evaluate potentially disabling or life-threatening pathologies.^1^ New neurology specific skills and competencies are expected at this transition. Residents start with varying levels of experience but need to quickly demonstrate competence in managing neurologic emergencies.^2^ According to the neurology-specific Accreditation Council for Graduate Medical Education (ACGME) milestones, residents are expected to be able to diagnose and manage neurologic emergencies, make a determination of death by neurologic criteria, perform lumbar punctures (LPs), and conduct patient-centered and family-centered communication.^3^ Despite the Institute of Medicine calling for increased attention to medical education research, the most cogent approach to addressing this transition has yet to be identified.^4^ The onus of designing curriculum and funding medical education research has fallen back onto training programs at academic medical centers.^5^

Studies show that simulation is highly effective at reinforcing knowledge and skills while improving learner confidence.^6^ High-fidelity simulations are increasingly incorporated into medical training because they allow residents to experience acute or potentially dangerous emergencies in a safe learning environment.^7^ Neurology physicians in training have reported educational benefit with acute ischemic stroke (AIS) simulation.^8^ In addition to refining these cognitive skills, simulation may also help with teaching communication and professionalism skills.^9^ A gap remains in neurology education for a comprehensive acute neurologic emergency simulation-based training curriculum for rising neurology residents.

In this article, we present the design and outcomes of an annual acute neurology simulation program with participation of residents from 2 separate neurology programs. We hypothesized that incoming neurology residents would (1) report improved confidence with managing acute neurologic emergencies, (2) cite a high degree of personal educational value with the simulation program, and (3) demonstrate improvement in their technical knowledge.

## Methods

### Curriculum Description

The high-fidelity simulation scenarios were part of a week-long neurology “bootcamp” held annually from 2019 to 2021 at our military training facility's Level 1 trauma center. Participants each year were the entire rising postgraduate year 2 class of neurology trainees in their first year as neurology trainees after internal medicine internship. Each year, the cohort of rising neurology residents from our program participated, with the final year also including the residents from a neighboring civilian institution, the University of Texas Health San Antonio. A simulation laboratory and staff were used to develop and execute simulations based on the ACGME neurology milestones.^3^ We created simulation scripts (available by contacting corresponding author), milestone checklist (see eAppendix 1 for the acute stroke checklist, links.lww.com/NE9/A5), and learning objectives (eAppendix 2, links.lww.com/NE9/A6) for each simulation. The neurologic emergency scenarios included AIS, brain death and delivering bad news, and status epilepticus (SE). The case scenario descriptions are provided as a supplemental file (eAppendix 3, links.lww.com/NE9/A7). Performing a LP was assessed as a standalone procedural skill. Skills education and scenario-based training were performed on a high-fidelity mannequin called the SimMan 3G (Laerdal, Norway), which is an advanced patient simulator that can display neurologic symptoms and physiologic findings. It features automatic physiologically linked drug recognition. LP training was performed on the Simulab LP Trainer, which support 3 varieties of life-like, ultrasound compatible, and replaceable tissues that mirror anatomic differences in the anatomy of the lumbar region in obese, geriatric, and normal patient populations.

### Evaluation Plan

On the first and last day of the course, participants completed the same prequestionnaires and postquestionnaires, respectively. Questionnaires required participants to self-rate their confidence on a Likert scale from 1 to 5 (1 = not at all confident and 5 = very confident) on the neurologic emergency topics covered. This method represents a more objective way to perform a learning needs assessment, an essential step to more specifically design educational activities to meet an endorsed gap.^10^

Questionnaires included 4 “yes” or “no” clinical questions regarding the simulations' acute interventions as a way to gauge technical clinical knowledge. For example, in the AIS simulation, these questions included identifying contraindications to tissue plasminogen activator (tPA): systolic blood pressure greater than 180 mm Hg, prior large ischemic stroke 3 months ago, current warfarin use with international normalized ratio of 1.8, and gastrointestinal bleed 30 days ago. For the SE simulation, this included whether clinical signs were consistent with SE: somnolent but arousable, right-gaze deviation, tonic posturing of upper extremities, hypotension, and bradycardia. For the brain death simulation, this included identifying contraindications: received Ativan 1 hour ago, febrile to 37.5°C, brain imaging demonstrates no pathology, and blood pressure 95/50 mm Hg.

After the prequestionnaire and before performing the simulations, the residents attended staff lectures on the relevant topics led by subspecialists from vascular neurology, neurocritical care, epilepsy, clinical neurophysiology, and palliative care. The palliative care specialists provided teaching regarding approaching end-of-life discussions and reviewing the 6-step SPIKES protocol for delivering bad news.^11^ At the end of the course, residents were also asked to report on a Likert scale (1 = not at all valuable and 5 = very valuable) and their perceived value of the simulations in their medical education.

Faculty observed the trainees performance of each skill and validated these skills using standardized scenario-specific checklists. At the time of course development, there was no standardized tool for individual validation of these skills; therefore, these assessment tools were adapted from widely available simulation instruction materials and internal expert opinion (eAppendix 1, links.lww.com/NE9/A5). The purpose of the checklists was to enable a systematic 2-way feedback session rather than give the impression of subjective academic scrutiny. The overall goal of the exercise and the “bootcamp” in general was to encourage participants to reach the same expected level of confidence and knowledge.

### Statistical Analysis

Statistical analysis was performed in IBM SPSS Statistics. Paired data were analyzed using a Friedman test for significance using a *p* value of 0.05. A Cohen *d*, or standardized mean difference, was also calculated to measure effect size. Multiple comparisons correction was not performed as this article reports on 3 simulations with separate sets of questions. If surveys were not correctly completed to allow for paired analysis, they were excluded from paired data analysis but still included in the descriptive statistics at each time point. Institutional review board review was not completed for this project because it was determined to be a quality improvement initiative by the Human Research Protections Office, and data collection did not include personally identifiable information. The views expressed here are those of the authors and do not reflect the official views or policy of the Department of Defense or its components.

### Data Availability

Anonymized data not published within this article will be made available by request from any qualified investigator.

## Results

Over 3 years, 15 rising neurology residents from 2 separate programs participated in these acute neurology simulations: 2 in 2019, 2 in 2020, and 11 in 2021. The first 2 years included only our military training facility's neurology trainees, and the final year included 4 from our institution and 7 a neighboring civilian program. Owing to the military-centric design of the SE simulation, only military facility residents (6 total) participated.

Self-reported confidence in simulation skills was low at baseline. Self-reported confidence in all acute neurology skills confidence improved after each simulation based on a predefined cut off of *p* < 0.05 (Table 1). For the AIS simulation, these skills included assessing for and identifying contraindications to tPA, identifying AIS, and identifying thrombectomy candidates. For the SE simulation, these skills included identifying clinical and electrographic SE and diagnosing and treating SE. For the brain death and delivering bad news simulation, these skills included identifying contraindications to and confounders of brain death diagnosis, performing the brain death examination, and delivering bad news to families. All the simulation skill changes had a large effect based on a Cohen *d* calculation (>0.8), except for identifying brain death confounders, which approached a medium effect. The LP simulation was not included in the skills confidence questionnaire. Technical knowledge (Table 2) also statistically improved with the stroke (*p* = 0.046) and brain death simulation (*p* = 0.039), but not the SE simulation (*p* = 0.296).

**Table 1:** Self-Reported Learner Confidence Before and After Acute Neurology Simulations

Simulation and clinical skills	Mean ± SD		*p* Value	Cohen *d*
	Pre	Post		
AIS simulation (n = 15)				
Assess for tPA	2.42 ± 1	3.82 ± 0.58	0.001	1.7
Identify AIS	3.17 ± 0.72	3.92 ± 0.67	0.005	1.1
Identify contraindications	2.67 ± 0.78	3.75 ± 0.45	0.002	1.7
Assess for thrombectomy	2.17 ± 0.94	3.67 ± 0.65	0.001	1.9
Status SE (n = 6)				
Identify clinical SE	2.20 ± 0.45	3.4 ± 0.55	0.046	2.4
Identify electrographic SE	1.3 ± 0.63	2.17 ± 0.75	0.025	1.3
Diagnose SE	2.0 ± 0.89	3.5 ± 0.55	0.025	2.0
Treat SE	1.67 ± 0.52	3.67 ± 0.52	0.014	3.8
Brain death simulation (n = 12)				
Identify brain death contraindications	2.18 ± 0.98	3.82 ± 0.60	0.002	2.0
Identify brain death confounders	2.17 ± 0.83	2.42 ± 0.67	0.002	0.4
Perform brain death examination	1.92 ± 0.79	3.83 ± 0.72	0.001	2.5
Explaining brain death	2.0 ± 0.74	4.0 ± 0.85	0.001	2.5

Abbreviations: AIS = acute ischemic stroke; SE = status epilepticus; tPA = tissue plasminogen activator.

Learners reported confidence on a 1–5 Likert scale. Significance report using the Friedman test.

**Table 2:** Correct Response (Y/N) Rate on Technical Knowledge Questions Per Simulation

	% Correct mean ± SD		*p* Value
	Pre	Post	
Stroke	64.6 ± 16.7	79.2 ± 20.9	0.046
Status epilepticus	75.0 ± 31.6	87.5 ± 13.7	0.296
Brain death	58.3 ± 12.3	75.0 ± 23.8	0.039

Satisfaction with the simulations was high. Participants reported an average educational value of 4.8 ± 0.4/5 (96%), 4.25 ± 1.0/5 (85%), and 4.7 ± 0.7/5 (94%) and for the AIS, SE, and brain death simulations, respectively.

## Discussion

This acute neurology simulation aided in the transition of new neurology residents by helping to teach and provide practice in the management of acute neurologic emergencies in a safe learning environment. This time of transition is anecdotally challenging and critical. Indeed, baseline confidence and knowledge for our learners were low. Our results suggest that confidence in performing specific skills across all 3 common neurologic emergency simulations increased including AIS, SE, and brain death. These skills are commonly experienced during on-call shifts, requiring independent thinking and quick decisions without immediate supervisory oversight.

The high perceived “value” scores indicate the utility of the simulation scenarios. Technical knowledge improved for the AIS and brain death emergencies, but not in the SE emergency. Although the average correct response rate did improve for the SE emergency, it may have lacked significance because of a lack of power. Civilian residents did not complete this scenario because of its battlefield setting. In addition, the average correct response rate before the SE simulation (75%) was similar the postsimulation response rate for the AIS (79%) and brain death (75%) simulations, suggesting a ceiling effect with high pre-existing knowledge among learners. Future iterations of the SE simulation should attempt to alter the difficulty of the technical clinical skill questions to assess for an intervention effect.

This study was limited by the self-reported nature of the questionnaires. These types of questions are prone to social desirability bias, wherein respondents may be inclined to respond in a socially acceptable way, such as ranking their confidence as low on precourse questionnaires because they believe it is expected.^12^ However, we argue that the combination of increased confidence, correct technical answer responses, and high value scores on an anonymous survey suggest a preponderance of the data from this study. Our program neither formally tracked individual participant test and simulation performance scores despite the use of the rubric nor solicited faculty commentary on perceived resident skill and knowledge as performed in other studies.^8^ This was intentionally designed to mitigate resident perceptions of any punitive measures, and therefore, mental or emotional stress at a time where there are already many new expectations and responsibilities.

This study is in-line with the evidence-based educational literature that simulation is a practical and high-yield method to educate graduate medical education trainees.^6,7^ It is of important that our study adds to the relatively sparse literature that simulations can be effectively pivoted to neurology education. Future studies should include collection of data on rubric accuracy, actual stroke response measures, in-service training examination scores, and even staff perceptions can be considered as longitudinal data points.

Neurology residency program directors and rising chief residents may recognize that new resident cohorts may have low confidence in neurologic clinical skills, heterogeneous technical knowledge, and generally be concerned about performance with indirect staff supervision. Our experience demonstrates that simulation programs strengthen the baseline knowledge and confidence of the neurologist in training. Because simulation training programs can be daunting to develop and implement, this article describes the process and tools required to successfully implement the curriculum. Ultimately, partnering with neighboring institutions may be synergetic solution for efficient execution.

We intend to continue this annual neurology simulation course with participation from our neighboring institution as local coronavirus disease 2019 pandemic precautions allow. Other neurology residency programs may consider incorporating similar simulation courses for their rising residents as a way of establishing a baseline content familiarity and improving resident confidence. More broadly, there is an opportunity to survey the neurology training community to better understand how residency programs use simulation in the transition to neurology-specific training and create a resource sharing platform that could benefit the field as a whole.

## Appendix Authors

**Table:**

Name	Location	Contribution
Zahari N. Tchopev, MD, MBA	Department of Neurology, Brooke Army Medical Center, Ft. Sam-Houston, TX	Drafting/revision of the manuscript for content, including medical writing for content; major role in the acquisition of data; study concept or design; analysis or interpretation of data
Alexis E. Nelson, MD	Department of Neurology, Brooke Army Medical Center, Ft. Sam-Houston, TX	Drafting/revision of the manuscript for content, including medical writing for content; Drafting/revision of the manuscript for content, including medical writing for content
John C. Hunninghake, MD	Department of Critical Care Medicine, Brooke Army Medical Center, Ft. Sam-Houston, TX	Drafting/revision of the manuscript for content, including medical writing for content
Kelsey Cacic, MD	Department of Neurology, Brooke Army Medical Center, Ft. Sam-Houston, TX	Drafting/revision of the manuscript for content, including medical writing for content, major role in the acquisition of data; study concept or design
Melissa K. Cook, MD	Department of Neurology, Brooke Army Medical Center, Ft. Sam-Houston, TX	Major role in the acquisition of data; study concept or design
Morgan C. Jordan, DO	Department of Neurology, Brooke Army Medical Center, Ft. Sam-Houston, TX	Drafting/revision of the manuscript for content, including medical writing for content; major role in the acquisition of data; study concept or design; analysis or interpretation of data

## Glossary

ACGME: Accreditation Council for Graduate Medical Education
AIS: acute ischemic stroke
LP: lumbar puncture
SE: status epilepticus
tPA: tissue plasminogen activator
